# Hyperspectral Imaging for Microcirculatory Assessment of Patients undergoing Transcatheter and Surgical Aortic Valve Replacement-a Prospective Observational Pilot Study

**DOI:** 10.1007/s12265-024-10573-z

**Published:** 2024-11-08

**Authors:** Maximilian Dietrich, Aycan Tayan, Tobias Hölle, Christian Nusshag, Anne-Christine Kapp, Christina Mertens, Alexander Studier-Fischer, Felix Nickel, Florian Leuschner, Markus Alexander Weigand, Matthias Karck, Christoph Lichtenstern, Rawa Arif, Dania Fischer

**Affiliations:** 1 Department of Anesthesiology, Heidelberg University, Medical Faculty Heidelberg, Im Neuenheimer Feld 420, 69120 Heidelberg, Germany; 2 Department of Nephrology, Heidelberg University Hospital, Heidelberg, Germany; 3 Center for Translational Biomedical Iron Research, Department of Pediatric Oncology, Immunology, and Hematology, University of Heidelberg, INF 350, 69120 Heidelberg, Germany; 4 Department of General, Visceral and Transplantation Surgery, Heidelberg University Hospital, Heidelberg, Germany; 5 Department of Internal Medicine III, University of Heidelberg, Im Neuenheimer Feld 410, 69120 Heidelberg, Germany; 6 Institute of Cardiac Surgery, University Hospital Heidelberg, Heidelberg, Germany

**Keywords:** Aortic valve replacement, Cardiopulmonary bypass, Microcirculation, Hyperspectral imaging, Capillary leak, Tissue oxygenation

## Abstract

This prospective, observational study evaluated Hyperspectral Imaging (HSI) to assess the effects of cardiac surgery and cardiopulmonary bypass (CPB) on microcirculation. 40 Patients with severe aortic stenosis were enrolled. 20 patients underwent transapical/transaxillary/transaortic aortic valve replacement (TAVR), 20 underwent an open surgical approach with CPB (SAVR). HSI was used to determine tissue oxygenation (StO_2_ and NIR), hemoglobin (THI) and water content (TWI) at the palm before/after surgery (T1/T2), and on the third postoperative day (T3). TAVR patients showed no significant changes of microcirculatory parameters during surgery. TWI significantly increased until T3. SAVR patients showed an increase of TWI and a decrease of THI, while StO_2_ and NIR remained unchanged at T2. In SAVR patients, StO_2_ and NIR correlated negatively with the duration of CPB and StO_2_ correlated with intraoperative urine output at T2. HSI was able to detect microcirculatory changes during cardiac surgery. CPB duration seemed to affect tissue oxygenation.

Clinical trial registration: (German Clinical Trial Register): DRKS00024765.

**Graphical Abstract:**

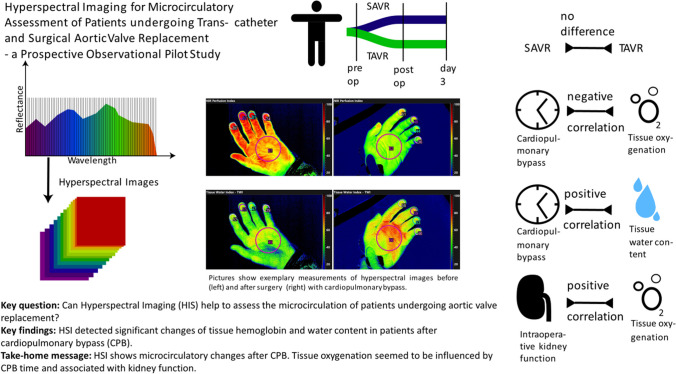

**Supplementary Information:**

The online version contains supplementary material available at 10.1007/s12265-024-10573-z.

## Background

Cardiopulmonary bypass (CPB) is a key component for performing complex cardiac surgical procedures requiring cardiac arrest. However, CPB leads in particular to disturbances of the cardiovascular system and coagulation, which increase with longer bypass duration [1]. Furthermore, CPB induces a release of proinflammatory mediators with systemic inflammatory response. De Backer et al. reported microcirculatory alterations associated with cardiac surgery using side-stream darkfield imaging [2]. In addition, decreased oxygenation of mucosa and skin was observed in response to CPB [3]. Since plasma levels of glycocalyx biomarkers increased after CPB and a correlation to microcirculatory alterations was found, glycocalyx shedding has been proposed as one underlying pathomechanism [4].

The macrocirculatory response to CPB shows a distributive shock pattern with vasodilatation and volume deficiency. The most severe manifestation is described as a vasoplegic syndrome with capillary leak similar to septic shock [1, 5]. Therefore, successful weaning from CPB often requires hemodynamic optimization combining fluid resuscitation with vasopressor and inotropic therapy [6]. Currently, hemodynamic therapy is mainly guided by macrocirculatory parameters and echocardiographic findings. Although the maintenance of adequate tissue perfusion is the ultimate goal of any hemodynamic therapy, this is currently not directly measured perioperatively due to the lack of practicable methods.

Hyperspectral imaging (HSI) uses the specific light absorbance of hemoglobin and water to determine non-invasively tissue oxygenation, hemoglobin and water content [7]. Especially the ability to measure tissue water content as a surrogate of capillary leakage could allow a comprehensive microcirculatory evaluation in distributive shock forms. This emerging technology was originally developed for wound diagnostics, but has also shown promising results for surgical resection planning and measurement of skin microcirculation in perioperative and critically ill patients [8–11]. In a porcine shock model, HSI indicated resuscitation effectivity based on tissue oxygenation and perfusion quality. The correlation of HSI skin and kidney measurements offers the reasoning to estimate organ oxygenation from skin monitoring [8].

The objective of this observational study was to assess the effects of cardiac surgery and CPB on tissue oxygenation, perfusion quality and water content in patients undergoing aortic valve replacement (AVR). The aim of this study was to evaluate the hemodynamic effects of AVR and the influence of an open surgical procedure with CPB on microcirculatory tissue oxygenation, perfusion quality and water content. Therefore, perioperative HSI measurements of the skin of the palm were performed in patients undergoing transapical, transaortic or transaxillary aortic valve replacement (TAVR) and patients undergoing surgical aortic valve replacement (SAVR) with CPB for the therapy of aortic stenosis.

## Methods

### Study Design and Settings

This prospective, observational pilot study was planned and performed by the Department of Anesthesiology in cooperation with the Department of Cardiac Surgery, as well as the Department of Cardiology, Angiology and Pneumology of the Heidelberg University Hospital, Germany. Patients were recruited from April 2021 to September 2022. The study has been performed in conformance with the ethical standards of the Declaration of Helsinki and its later amendments. The study was registered at the German Clinical Trial Register (DRKS00024765). The reporting of the study adheres to the STROBE guidelines [12].

### Ethics

Ethical approval for this study (reference number S-128/2021) was provided by the Ethical Committee of the Medical Faculty of Heidelberg University, Alte Glockengießerei 11/1, 69,115 Heidelberg, Germany (Chairperson Prof. Dr. med. Dr. h.c. Thomas Strowitzki) on 04 March 2021.

### Participants

Patients with severe aortic stenosis with an indication for AVR were enrolled, of which 20 patients (9 male (45%) / 11 female (55%)) were planned for transapical/transaxillary/transaortic AVR and 20 (11 male (55%) / 9 female (45%)) were planned for an open surgical approach with CPB (SAVR). The decision between TAVR and SAVR was made independently of this study by the interdisciplinary heart team before recruitment as recommended by both current European and American guidelines for the management of valvular heart disease. The patients were recruited on hospital admission and informed consent to participate was obtained. Inclusion criteria were age ≥ 18 years, signed informed consent, elective AVR for the therapy of an aortic stenosis. Exclusion criteria were AVR for the treatment of aortic regurgitation, refusal of participation, infectious viral diseases (HBV, HCV, HIV, COVID-19) or unknown COVID-19 status, chronic kidney failure with a glomerular filtration rate (GFR) < 15 ml/min/m^2^ or need for dialysis.

### AVR Procedures

SAVR patients received predominantly biological prostheses and few mechanical prostheses (Edwards Perimount Magna Ease, Edwards Inspiris, St. Jude Medical Aortic bileaflet, Perceval S) with the specific type and size determined during the procedure. All SAVR procedures were performed using CPB. TAVR patients received balloon expandable prostheses (Edwards Sapien 3 and Sapien 3 Ultra), predominantly via transapical access (80%) with some patients undergoing left transaxillary (10%) or transaortic (10%) access receiving a self-expandable protheses (Medtronic Evolut R). All procedures were performed by senior cardiac surgeons.

### Hyperspectral Imaging

The TIVITA® Tissue System (Diaspective Vision GmbH, Am Salzhaff, Germany), is a novel diagnostic tool, that measures the specific light reflection and absorbtion. The individual spectral characteristics of water, hemoglobin and desoxyhemoglobin allow for quantification of these tissue components. For each measurement a light is emitted and the reflected light is detected. This takes only a couple of seconds and provides a non-invasive diagnostic way, doesn’t use ionizing radiation and provides four different parameters:

- Tissue oxygenation (StO_2_, wavelength range: 500–650 and 700–815 nm): oxygen saturation of the hemoglobin in the superficial capillary system (penetration depth up to 1 mm), values are presented in percent (0–100);
- NIR perfusion index (NIR, wavelength range: 655–735 and 825–925 nm): oxygen saturation of the hemoglobin in the capillary system of deeper tissue layers (penetration depth up to 4–6 mm), values are presented in an arbitrary scale from 0–100;
- Tissue hemoglobin index (THI, wavelength range: 530–590 and 785–825 nm): distribution of deoxygenated and oxygenated hemoglobin in the measured tissue (penetration depth up to 1–3 mm), values are presented in an arbitrary scale from 0–100;
- Tissue water index (TWI, wavelength range: 880–900 and 955–980 nm): relative tissue water content (penetration depth up to 1–3 mm), values are presented in an arbitrary scale from 0–100.

The underlying physical principles, technical specifications and algorithms of the TIVITA® Tissue System have been reported in detail by Holmer et al.[7] Measurements were taken before surgery (T1), directly after surgery (T2) and on the third postoperative day. The TIVITA® Tissue System was applied in compliance with instructions of the manufacturer. For each analysis a circular region of 70 units on the palm was selected (the circle crossing the metacarpophalangeal joint 3–4). To prevent influence on clinical therapy, the measurements were not made available to the treating physicians.

### Anesthesia and Hemodynamic Management

All implantation procedures were performed in general anesthesia using propofol, sevoflurane and sufentanil/remifentanil. The treating anesthesiologist determined the hemodynamic therapy during the procedure. After the procedure, the patients were admitted to the intensive care unit of the Department of Cardiothoracic Surgery.

### Clinical Data

Demographic data, comorbidity, medication and data concerning the procedure, blood loss, administered fluids, vasoactive-inotropic medication, and anesthetics were documented. Vasopressor dependence and inotropic support were summarized and analyzed using the vasoactive-inotropic score (VIS) [13, 14]:

$$VIS=dopamine\;dose\;(\mu g/kg/min)+dobutamine\;dose\;(\mu g/kg/min)+100\times\;epinephrine\;dose\;(\mu g/kg/min)+100\times\;norepinephrine\;dose\;(\mu g/kg/min)+\text{10,000}\times\;vasopressin\;dose\;(U/kg/min)+10\times\;milrinone\;dose\;(\mu g/kg/min)$$

Blood samples and hemodynamic parameters were collected in parallel to the respective HSI measurements.

### Primary Outcome

The primary outcome parameters were the perioperative changes of HSI derived tissue oxygenation StO_2_ and NIR, hemoglobin (THI) and water content (TWI) of the palm from preoperative (T1) to postoperative after SAVR and TAVR (T2).

### Secondary Outcome

Secondary endpoints were the HSI derived tissue oxygenation StO_2_ and NIR, hemoglobin (THI) and water content (TWI) of the palm on the third postoperative day (T3). Further, the correlations of the HSI parameters StO_2_, NIR, THI and TWI to clinical variables like duration of CPB, fluid balance, intraoperative urine output, hemoglobin and vasopressor support were assessed at the end of surgery (T2).

### Statistical Methods

Descriptive statistics was performed on all relevant data. Mean, median, standard deviation, inter quartile range (IQR), min and max values were calculated. Comparison of metric values between groups was performed with a Mann–Whitney-U-Test, comparison of change over time was analyzed with a Friedman-Test. Categorical values were analyzed using a Chi-Squared-Test. Spearman-Test was used for correlation analyses. Level of significance was set at 0.05. Metric parameters are reported as median and IQR, categorical parameters are reported as absolute and relative frequencies. Statistical analysis was performed with SPSS version 29.0.

## Results

### Baseline Characteristics

Baseline characteristics showed a difference in age and weight between the two groups, with the SAVR patients being the younger and heavier patients. The duration of surgery was significantly shorter in the TAVR groups. ICU stay and hospital stay were comparable between the groups. Two patients died in the TAVR group during the hospital stay, notably, these patients weren´t the patients classified as ASA V. All patient characteristics are presented in Table 1.

**Table 1:** Baseline characteristics of the two cohorts. Patients received an aortic valve replacement either with cardiopulmonary bypass (SAVR) or with transcatheter technique (TAVR). Comparison between the two groups was perfomed with Mann–Whitney-U-Test for metric values and with a Chi-Squared-Test for categorical values. Significant values are written in bold characters. ASA: American Society of Anesthesiologists physical status classification; PAD peripheral arterial disease; DM diabetes mellitus; CAD coronary artery disease; LD lung disease; AHT arterial hypertension; CKD chronic kidney disease; ICU: Intensive care unit; AKI: Acute kidney injury; cm: Centimeters; kg: Kilogram; min: Minutes; d: days

		SAVR	TAVR	
Age		69 (62; 73.5)	80 (75.5; 84.5)	< 0.001
Height [cm]		170.5 (163.5; 178.5)	166.5 (159.5; 170.5)	0.056
Weight [kg]		84 (71.25; 99.5)	73.5 (65.5; 80)	0.04
ASA-classification	unclassified	1 (5%)	0 (0%)	0.56
	II	1 (5%)	0 (0%)	
	III	8 (40%)	7 (35%)	
	IV	10 (50%)	11 (55%)	
	V	0 (0%)	2 (10%)	
Comorbidities				
	PAD	8 (40%)	16 (80%)	0.01
	DM	9 (45%)	12 (60%)	0.342
	CAD	15 (75%)	19 (95%)	0.077
	LD	5 (25%)	13 (65%)	0.011
	Adipositas	10 (50%)	6 (30%)	0.197
	AHT	16 (80%)	19 (95%)	0.151
	CKD	3 (15%)	8 (40%)	0.077
Pre op Echo				
	Area [cm^2^]	0.8 (0.6; 0.9)	0.78 (0.6; 0.8)	0.283
	EF [%]	60 (55; 60)	54.5 (37.5; 55)	0.076
EuroSCORE		8 (5; 10)	12 (11; 14)	< 0.001
Duration of Surgery [min]		242 (195; 307.5)	74 (61.5; 91)	< 0.001
Clamping time [min]		87.5 (77; 116.5)		
Reperfusion time [min]		27 (21.5; 35)		
Cardiopulmonary bypass time [min]		122.5 (109; 165.5)		
ICU stay [d]		2 (2; 4)	2 (2; 3)	0.38
Hospital stay [d]		13 (11.5; 16)	13 (10; 20)	0.792
Intrahospital mortality		1 (5%)	2 (10%)	0.5
AKI		3 (5%)	5 (25%)	0.69

### Hemodynamic Changes After SAVR and TAVR

**Fig. 1:**
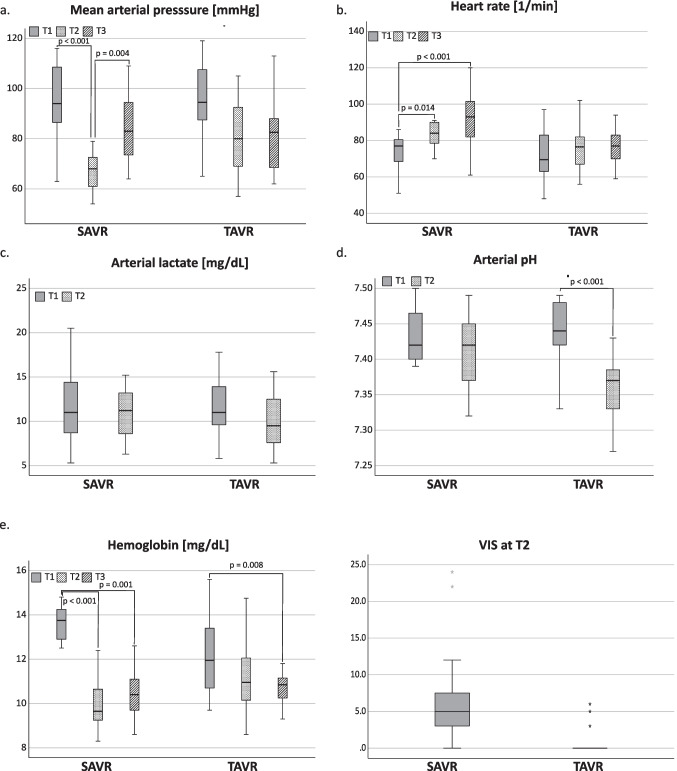
Change over time of hemodynamic parameters. Patients received an aortic valve replacement either in an open surgical procedure with cardiopulmonary bypass (SAVR) or with transcatheter technique (TAVR). Comparison was performed over the course of time, not between the individual groups. Measurements were conducted before surgery (T1), after surgery (T2) and on the third postoperative day (T3). VIS: Vasoactive Inotropic Score; mmHg: Milimetre of mercury; 1/min: Beats per minute; mg/dL: Miligram per decilitre; stars indicate outliers

Compared to baseline, TAVR patients showed a nonsignificant drop in the mean arterial pressure after the surgery. The decrease in pH, even though statistically significant, is still within the physiological range. Other major hemodynamic parameters did not differ from baseline. SAVR patients on the other hand showed major changes in hemodynamic parameters. Compared to baseline, AVR patients had a significant increase in heartrate and significantly lower mean arterial pressure after surgery. While the mean arterial pressure recovered, heart rate remained significantly higher compared to baseline. Hemoglobin level was significantly lower after surgery in the AVR group compared to baseline, and lower in both groups 3 days after surgery. It should also be noted that all patients who underwent SAVR required vasopressor support at T2, whereas only 4 patients in the TAVR group needed vasopressors. Hemodynamic variables are shown in Fig. 1.

### Microcirculatory Changes After SAVR and TAVR

**Fig. 2:**
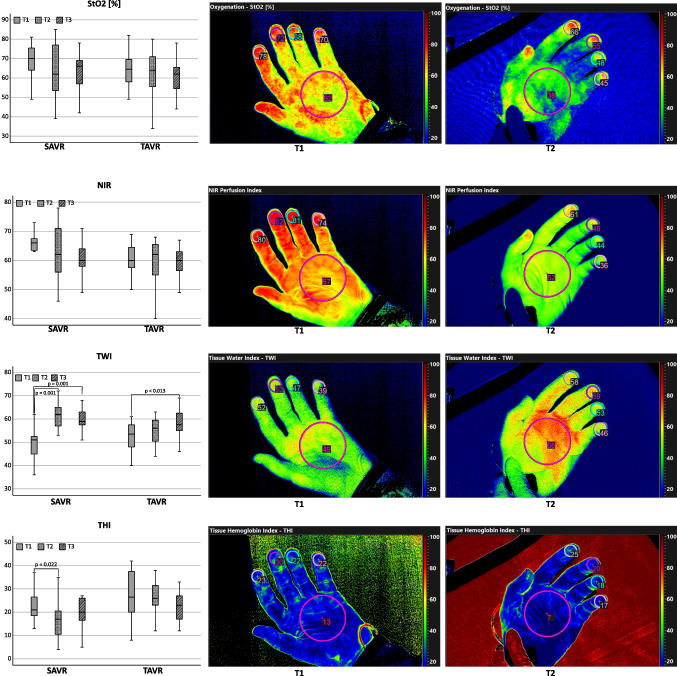
Change over time of hyperspectral images in patients receiving aortic valve replacement. Patients received an aortic valve replacement either in an open surgical procedure with cardiopulmonary bypass (SAVR) or with transcatheter technique (TAVR). Depicted are tissue oxygenation index in [%] (StO2), near infrared spectroscopy index (arbitrary index) (NIR), tissue water index (arbitrary index) (TWI) and tissue hemoglobin index (arbitrary index) (THI). Measurements were conducted before surgery (T1), after surgery (T2) and on the third postoperative day (T3). Pictures show exemplary measurements of patients receiving SAVR before(T1) and after surgery (T2)

TAVR patients showed no significant change of microcirculatory parameters after surgery. StO_2_ and NIR stayed close to baseline values. TWI non-significantly increased after surgery and further increased until the third postoperative day, which was significant compared to baseline. SAVR patients on the other hand showed a major increase in TWI after surgery with CPB (T2) as well as a decrease in THI: However, while the THI returned to the baseline level, the TWI remained elevated at T3. NIR and StO_2_ showed no significant alteration after surgery with CPB (T2). HSI measurements and change over time for both groups are depicted in Fig. 2.

### Correlation of HSI Parameters and Clinical Variables After SAVR with CPB

**Fig. 3:**
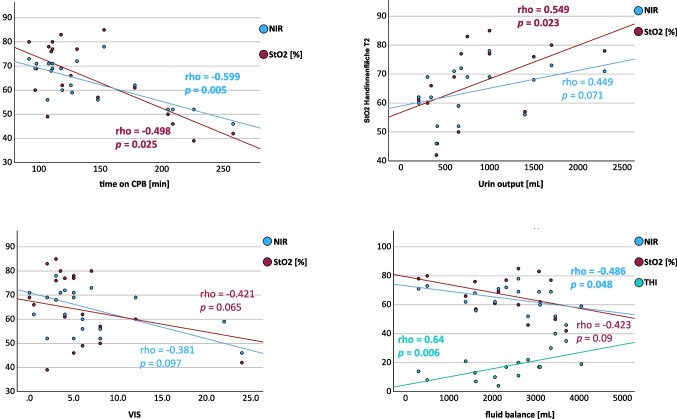
Correlation of hyperspectral measurements (HSI) after surgical aortic valve replacement with cardiopulmonary bypass duration, vasoactive-inotropic score, fluid balance and urine output. HSI values are shown on x-axis on a scale from 0 to 100. Figures show correlation between HSI and time on cardiopulmonary bypass (CPB), HSI and urinary output, vasoactive inotropic score (VIS) and HSI and fluid balance. Min: Minutes; mL: Mililitres

Further analysis of the influence of CPB showed a significant correlation of tissue oxygenation and bypass time. StO_2_ correlated with a coefficient of *p* = -0.498 (*p*= 0.025) and the deeper layer tissue oxygenation NIR with a coefficient of *p* = -0.599 (*p* = 0.005). Fluid balance after surgery also negatively correlated with NIR (*p* = -0.486; *p* = 0.048), the correlation with StO_2_, however did not reach statistical significance (*p* = -0.423; *p* = 0.09). Fluid balance also correlated with postoperative THI values (*p* = 0.64; *p* = 0.006). Urine output, as a sign of organ function, correlated with StO_2_ at T2 (*p* = 0.549; *p* = 0.023), the correlation with NIR however, did not reach significance (*p* = 0.449; *p* = 0.071). A correlation of VIS and tissue oxygenation showed a trend, but did not reach significance for both StO_2_ (*p* = -0.421, *p* = 0.065) and NIR (*p* = -0.381; *p* = 0.097). THI and hemoglobin level did not significantly correlate at T2 in patients undergoing SAVR. Correlation analyses of SAVR patients are shown in Fig. 3.

### Correlation of Hemodynamic and HSI Parameters after TAVR

In patients receiving TAVR there was no significant correlation between HSI and hemodynamic parameters. However, it has to be mentioned that TAVR itself did not alter macrohemodynamic parameters after surgery compared to baseline. Hemoglobin levels correlated with THI at T2 (*p* = 0.593; *p* = 0.006) in patients undergoing TAVR.

## Discussion

One of the primary challenges of the anesthesiologist in cardiac surgery is perioperative hemodynamic management. Until today, hemodynamic therapy is mainly controlled by macrocirculatory target parameters such as blood pressure and cardiac output, although the actual underlying goal is to maintain organ and tissue oxygenation and perfusion. Unfortunately, up to now no direct, easy to perform, bedside measurement technique for monitoring of tissue microcirculation is available in clinical routine. Cerebral oxygen saturation measurement by near infrared spectroscopy (NIRS) is the only established method for high-risk interventions. However, since the brain as a central organ is still perfused while other more peripheral organs are already underperfused, cerebral NIRS does not seem to be a suitable therapy target to maintain a good tissue perfusion globally [15].

In this study, we evaluated HSI for perioperative microcirculatory evaluation of the skin in patients undergoing TAVR and patients undergoing SAVR for the therapy of an aortic stenosis. Patients undergoing TAVR demonstrated a significantly higher prevalence of peripheral artery disease as a comorbidity. We believe this condition may substantially influence HSI skin measurements, particularly those related to tissue oxygenation. Consequently, we have refrained from making direct comparisons of microcirculatory HSI measurements between the two groups. TAVR did not have a significant influence neither on macrohemodynamic parameters, tissue oxygenation nor on tissue water content comparing the pre- and directly postoperative measurement. On the other hand, SAVR led to a strong increase of tissue water content and decrease of tissue hemoglobin content. In particular, the increase of tissue water content could potentially be explained by the inflammatory effects of CPB on endothelial function with consecutive capillary leakage [4, 16]. The hemodynamic effects of valve replacement per se seem to be secondary as there was no strong increase of tissue water content during TAVR. Transfemoral access for transcatheter procedures is typically favored over non-transfemoral access in our institution. In this study, non-transfemoral access was employed in patients with anatomical conditions that contraindicated transfemoral TAVR. Importantly, the choice of access route is not expected to significantly influence skin microcirculation or HSI measurements, suggesting that our results are transferable to transfemoral TAVR procedures. However, the greater extent of the surgical trauma of the open surgical procedure and intraoperatively administered fluids must also be considered in the interpretation of these findings. In patients undergoing pancreatic surgery TWI rose during the operation and a stronger increase of TWI was associated with higher blood loss, glycocalyx damage and duration of surgery. In a porcine model of hemorrhage TWI increased in response to fluid resuscitation [8, 17]. The observations of the present study suggest that HSI is sufficiently sensitive to monitor perioperative changes in tissue water content. To date, there is no established clinical method to measure tissue edema non-invasively at the bedside in real time. However, the clinical relevance of perioperative TWI kinetics in relation to clinical outcomes and how these can be influenced by hemodynamic therapy need to be evaluated in future studies. The observations of the present study suggest that HSI is sufficiently sensitive to monitor perioperative changes in tissue water content.

THI measures the content of both oxygenated and deoxygenated hemoglobin in the tissue. In previous publications, elevated THI of the skin has been described in association with increased mortality in septic patients, venous congestion in surgical flaps, and as a consequence of high-dose catecholamine therapy in hemorrhagic shock [17]. The decrease observed here may be related to the perioperative decrease in blood hemoglobin levels. However, we only observed a significant correlation between THI and hemoglobin level after TAVR, but not after SAVR with CPB. This fits with observations in patients undergoing pancreatic surgery [8]. A THI decrease has also been reported in cases of reduced perfusion due to arterial occlusion [18, 19]. In conclusion, THI seems to underlie multifactorial influences and needs to be interpreted in context to the clinical situation.

HSI parameters for tissue oxygenation (StO_2_, NIR) did not change significantly during both open surgical and transcatheter AVR. This could be due to the fact that all patients had stable hemodynamic conditions during the measurements and none of the patients had a manifest state of shock. However, correlation analysis revealed a significant association of StO_2_ and NIR to the duration of CPB and relevant clinical variables. Longer bypass time was accompanied by poorer postoperative tissue oxygenation. A study using sublingual sidestream darkfield imaging for microcirculatory evaluation also reported a significant deterioration of microcirculatory perfusion in response to CPB [20]. Further, the StO_2_ and NIR at the end of surgery correlated positively with intraoperative urine output. Catecholamine requirements at the end of surgery showed a trend towards negative correlation with HSI tissue oxygenation markers.

Both observations are in line with the results of a porcine study, that showed a high correlation of skin and kidney oxygenation as a surrogate marker of perfusion measured with HSI and a negative impact of high-dose norepinephrine on tissue oxygenation in hemorrhagic shock [17]. Even though there was no change in tissue oxygenation markers during surgery in the overall cohort, the pathomechanistically meaningful correlations to relevant established haemodynamic variables indicate that tissue perfusion is reflected by the HSI parameters. This also points to the assumption that the correlation between organ and skin microcirculation already shown in a porcine model is also present in perioperative patients. Further, Kuhlmann et al. reported a strong association of HSI tissue oxygenation markers with norepinephrine support and blood lactate levels in critically ill patients with COVID-19 [21].

This study was an observational pilot study to assess the use of hyperspectral imaging to visualize the microcirculatory effects of cardiac surgery and CPB. Several limitations need to be considered. The relation of microcirculatory alterations to postoperative complications or clinical outcomes could not be investigated due to the small number of included patients. Furthermore, the patients and extent of trauma of TAVR and open surgical AVR procedure clearly differ, so we refrained from performing a comparative analysis of the two procedures with respect to the microcirculatory effects.

## Conclusion

HSI was able to detect significant changes of tissue hemoglobin and water content in patients undergoing open surgical AVR with CPB. AVR and CPB did not significantly influence tissue oxygenation as all patients were measured in a stable macrohemodynamic situation. Nevertheless, tissue oxygenation seemed to be influenced by bypass time and to be associated with intraoperative kidney function. The results of this study suggest that HSI is a promising technique to allow perioperative bedside microcirculatory evaluation in patients undergoing cardiac surgery. HSI should be evaluated in larger cohorts in relation to postoperative complications such as delirium and acute renal failure. To date, there is no objective technique to measure tissue perfusion and water content non-invasively at the bedside. HSI could potentially fill this diagnostic gap, allowing hemodynamic therapy and interventions to be guided by microcirculatory parameters. Interventional trials are necessary to evaluate the potential of HSI tissue oxygenation markers as target parameters for hemodynamic management.

## Supplementary Information

Below is the link to the electronic supplementary material.

Supplementary file1 (PDF 137 KB)

## Data Availability

MD, AT, TH and DF had full access to all the data in the study and take responsibility for its integrity and the data analysis. The datasets used and analyzed during the current study are available from the corresponding author on reasonable request.
