# Supplementary figures and images for: Hyperspectral Imaging for Microcirculatory Assessment of Patients undergoing Transcatheter and Surgical Aortic Valve Replacement-a Prospective Observational Pilot Study
Source: J Cardiovasc Transl Res. 2024 Nov 8;18(2):295–304. doi: 10.1007/s12265-024-10573-z (PMC12043735; doi:10.1007/s12265-024-10573-z)

a.

O<sub>2</sub> [mmHg]

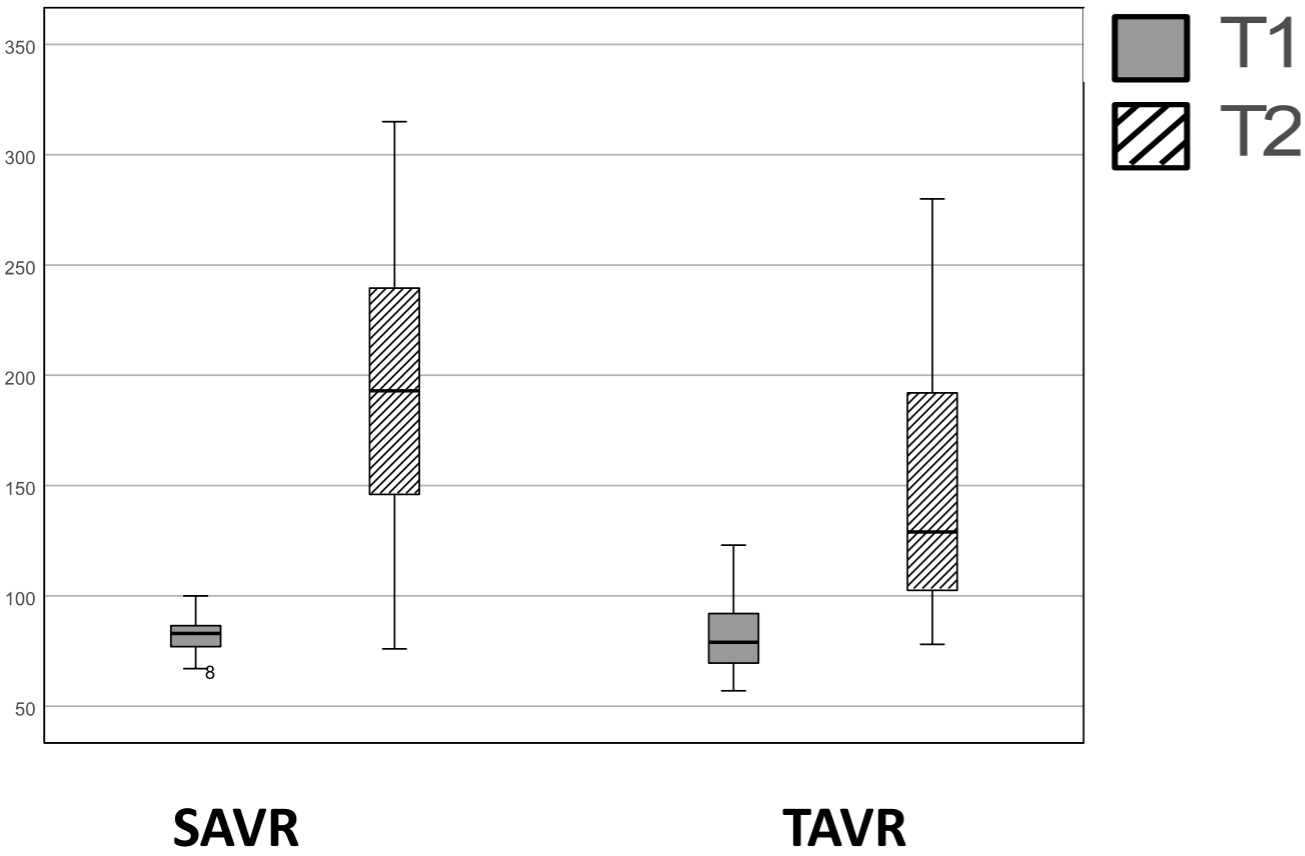

b.

CO<sub>2</sub> [mmHg]

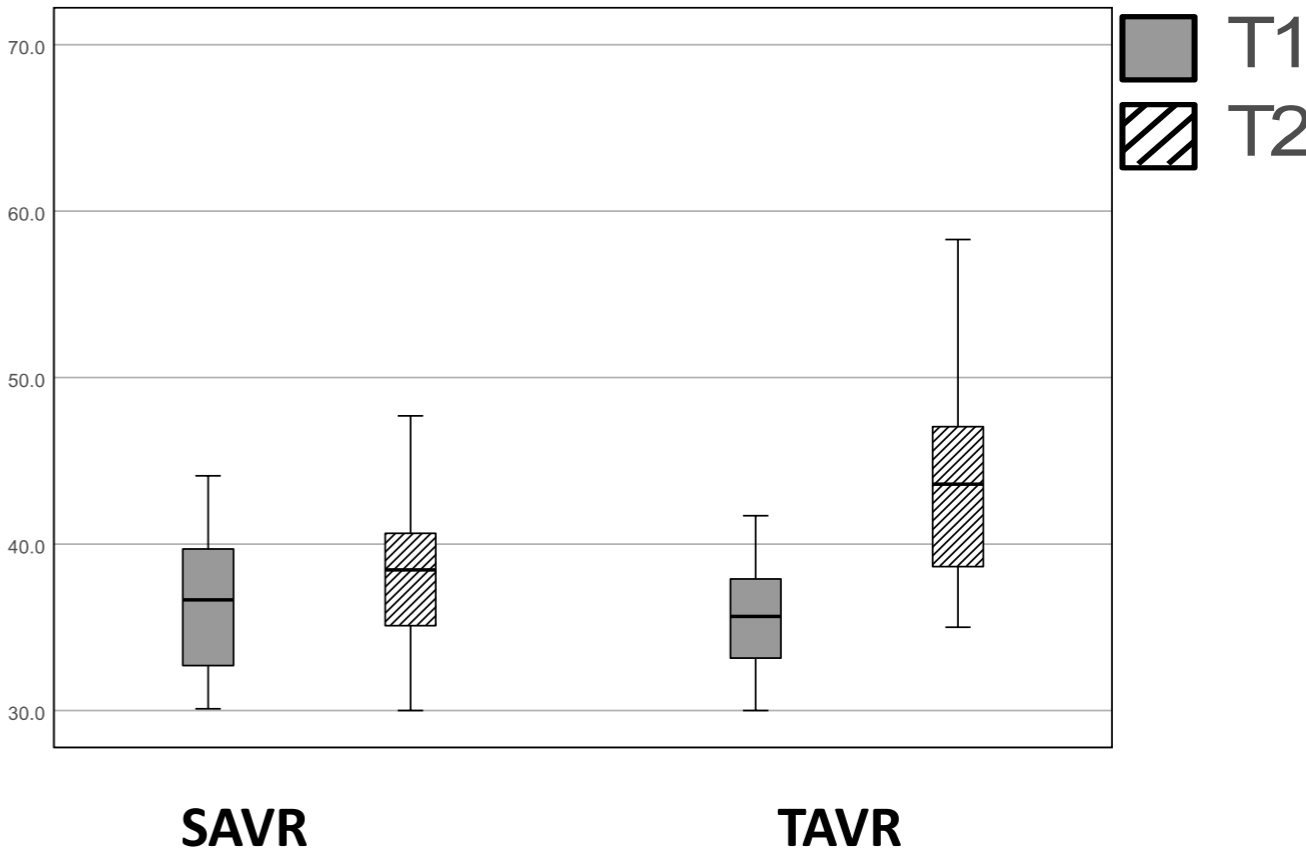

c.

Bicarbonate [mmol/L]

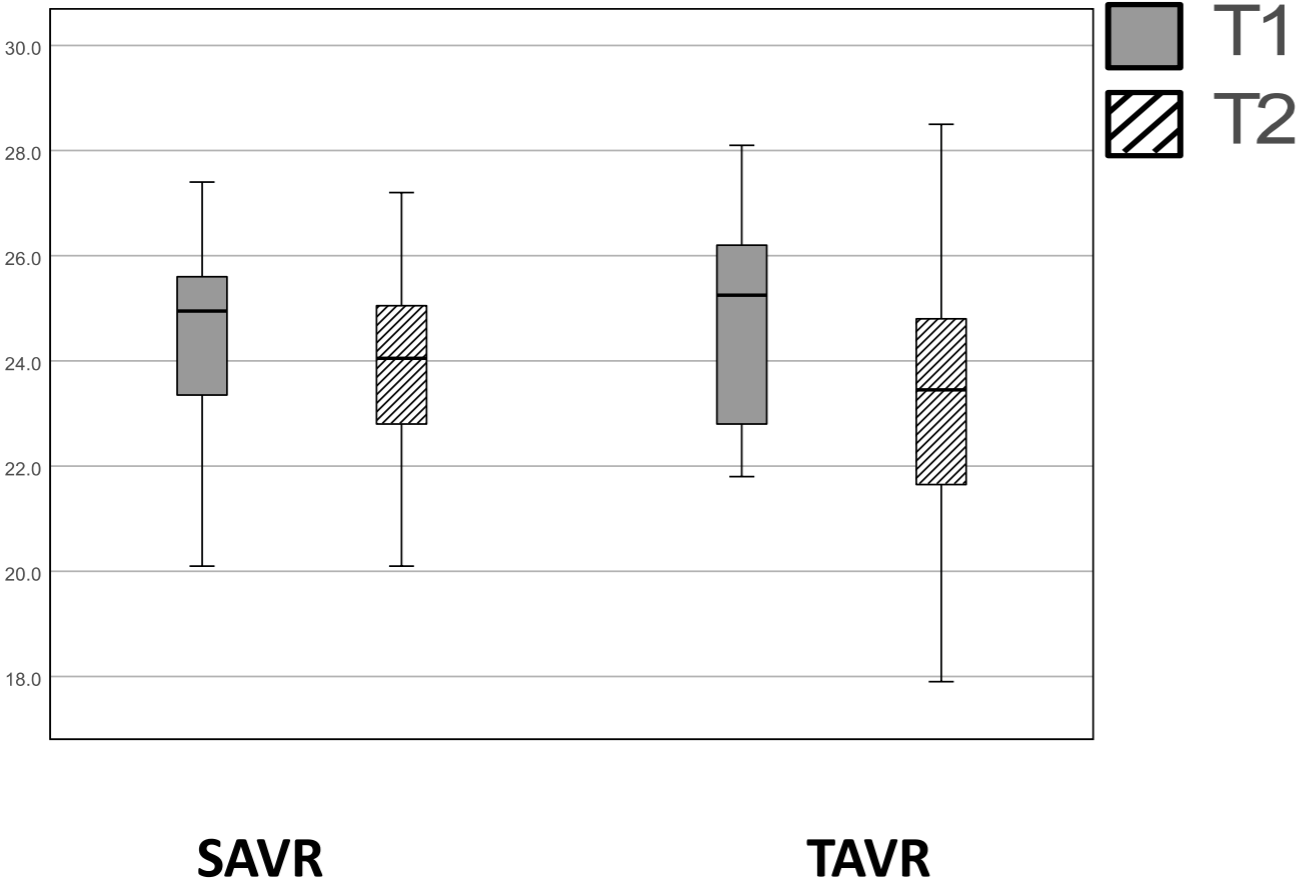

d.

Base excess [mmol/L]

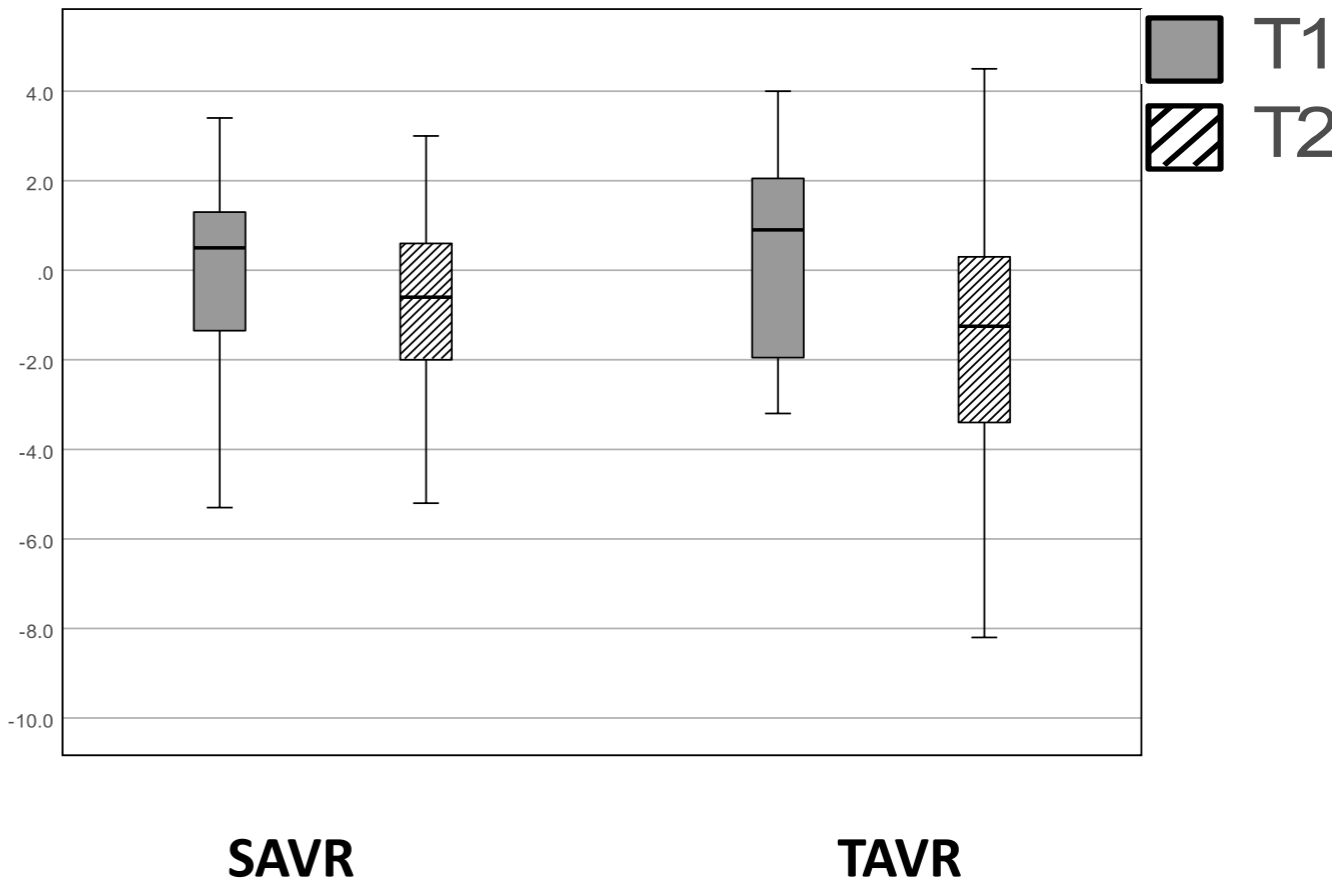

e.

Lactate [mg/dL]

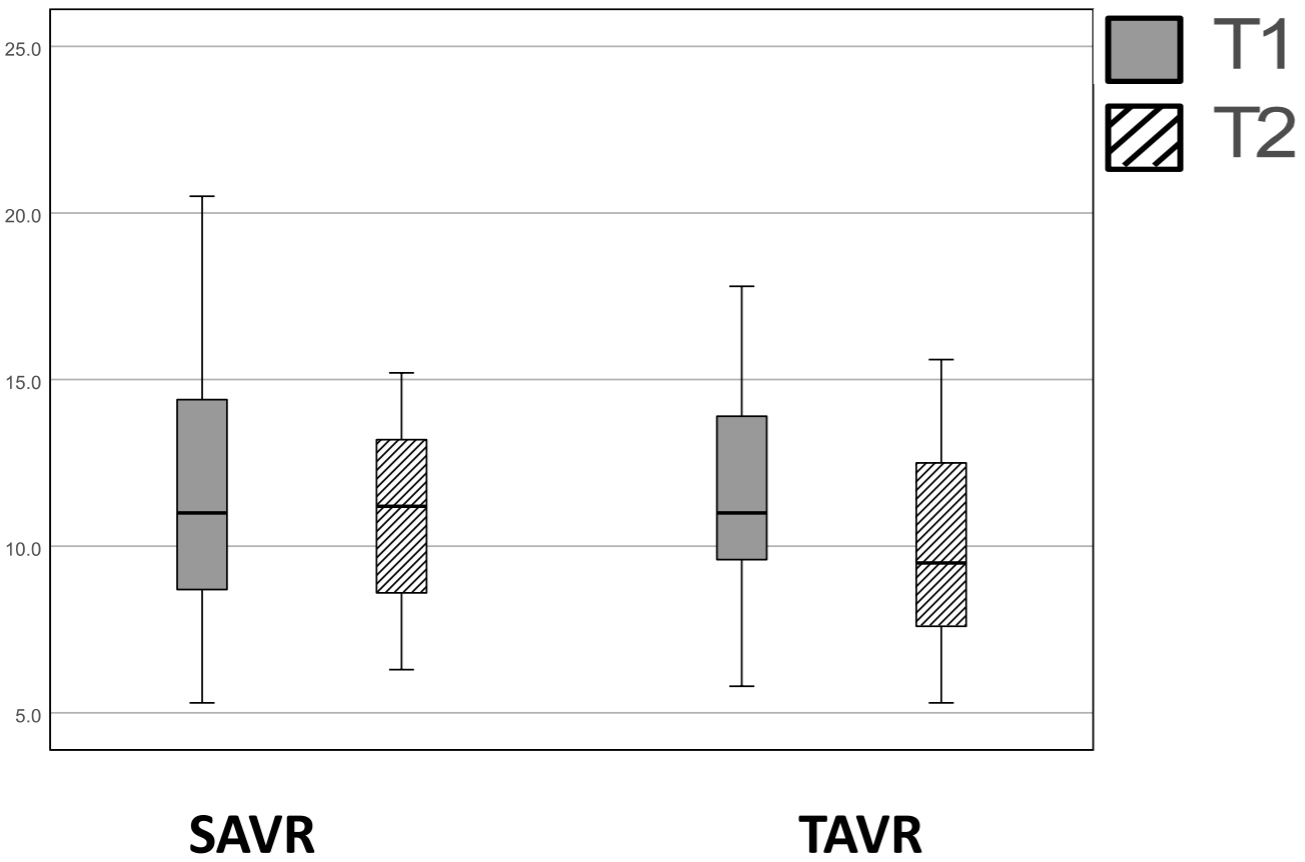

pH

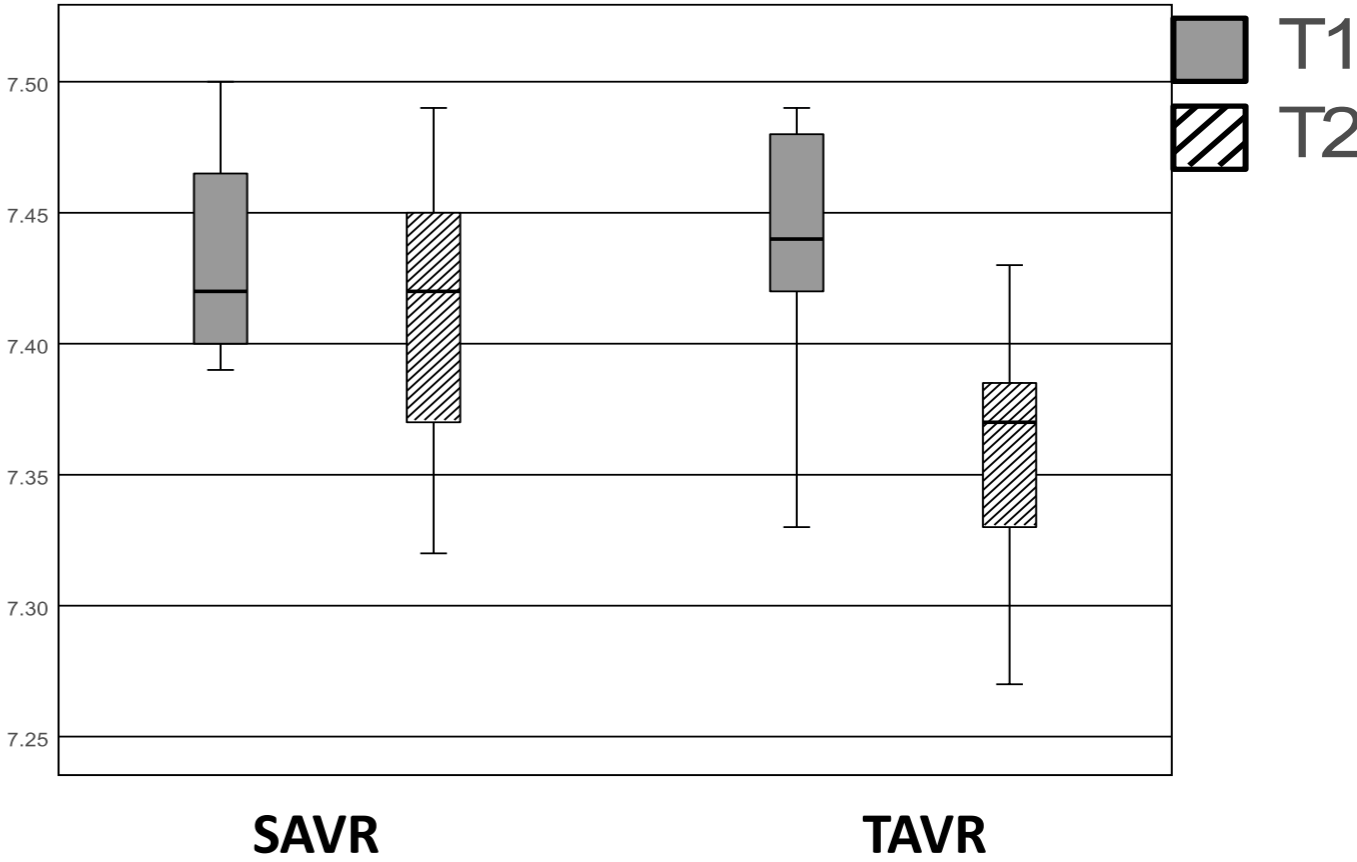

Supplement: Supplementary file 1 — Supplementary file1 (PDF 137 KB) [file 12265_2024_10573_MOESM1_ESM.pdf]
